# Microsphere integrated microfluidic disk: synergy of two techniques for rapid and ultrasensitive dengue detection

**DOI:** 10.1038/srep16485

**Published:** 2015-11-09

**Authors:** Samira Hosseini, Mohammad M. Aeinehvand, Shah M. Uddin, Abderazak Benzina, Hussin A. Rothan, Rohana Yusof, Leo H. Koole, Marc J. Madou, Ivan Djordjevic, Fatimah Ibrahim

**Affiliations:** 1 Department of Biomedical Engineering, Faculty of Engineering, University of Malaya, Kuala Lumpur, 50603, Malaysia; 2 Center for Innovation in Medical Engineering, Faculty of Engineering, University of Malaya, Kuala Lumpur, 50603, Malaysia; 3 Faculty of Health, Medicine and Life Sciences, Maastricht University, the Netherlands; 4 Department of Molecular Medicine, Faculty of Medicine, University of Malaya, 50603, Kuala Lumpur, Malaysia; 5 Department of Biomedical Engineering, University of California, Irvine, 92697, United States; 6 Department of Mechanical and Aerospace Engineering, University of California, Irvine, 92697, United States

## Abstract

The application of microfluidic devices in diagnostic systems is well-established in contemporary research. Large specific surface area of microspheres, on the other hand, has secured an important position for their use in bioanalytical assays. Herein, we report a combination of microspheres and microfluidic disk in a unique hybrid platform for highly sensitive and selective detection of dengue virus. Surface engineered polymethacrylate microspheres with carefully designed functional groups facilitate biorecognition in a multitude manner. In order to maximize the utility of the microspheres’ specific surface area in biomolecular interaction, the microfluidic disk was equipped with a micromixing system. The mixing mechanism (microballoon mixing) enhances the number of molecular encounters between spheres and target analyte by accessing the entire sample volume more effectively, which subsequently results in signal amplification. Significant reduction of incubation time along with considerable lower detection limits were the prime motivations for the integration of microspheres inside the microfluidic disk. Lengthy incubations of routine analytical assays were reduced from 2 hours to 5 minutes while developed system successfully detected a few units of dengue virus. Obtained results make this hybrid microsphere-microfluidic approach to dengue detection a promising avenue for early detection of this fatal illness.

Application of micro- and nanospheres in biomedical domain is ubiquitous as such spherical platforms address some of the major limitations of current bioassay practices^1^,^2^. Some key advantages of the micro/nano spheres over other bioreceptor platforms are: (i) amenable to screening and multiplexing; (ii) enhanced receptor conjugation due to their significantly larger surface area compared to two dimensional (2D) platforms; and (iii) spatial freedom to query the entire sample volume thus promoting more effective analyte-substrate interaction^2^,^3^,^4^,^5^,^6^,^7^,^8^,^9^. In particular, micro and nanospheres have attracted increasing attention in biosensor research as enhancers of biorecognition and have frequently been used for detection of several categories of targeted biomolecules^2^,^10^,^11^,^12^,^13^,^14^,^15^. Metallic particles such as silver nanoparticles (AgNPs), gold nanoparticles (AuNPs), paramagnetic microspheres, as well as polymeric microspheres became standard in analytical applications^2^,^4^,^16^,^17^. An interesting newer development is the integration of such microspheres inside microfluidic lab-on-a-chip (LOC) devices^18^,^19^.

A LOC device is a portable, low cost microfluidic platform designed to automate one or more analysis steps such as mixing, separation and sedimentation into one monolithic device^20^,^21^,^22^. Although LOC devices facilitate various chemical procedures, due to the importance of health care issues, their applications are mainly geared towards point of care (POC) diagnostics. Such devices act as a miniaturized laboratory system that can be used in remote and/or rural areas for detection of multiple viruses without human involvement, which, in turn, makes the detection procedure of fatal diseases safer for laboratory technicians^23^,^24^,^25^,^26^. Centrifugal microfluidics is a LOC technology in which a simple motor generates pseudo forces by spinning a compact disk (CD)-like microfluidic platform to propel liquids within microchannels and microchambers embedded in the disk^27^. Centrifugal microfluidics does not involve expensive, external pumps and complicated plastic tubing, typically required for the automation of the complicated fluidic manipulations^28^. Micromixing in microfluidic platforms, in general, refers to the set of techniques that are used to homogenize reagents or to increase the reaction rate between sensor’s surface and the target analyte^29^,^30^,^31^,^32^. Particularly, in microfluidic disks, micromixing is an essential fluidic step to enhance the rate of bioreactions and to reduce the incubation time of bioanalytical assays^33^,^34^. Flow reciprocation through the passive generation of pneumatic energy and shaking by sudden reversing the rotation direction of the fluidic platform are two mixing techniques that have been used in microfluidic disks^33^,^35^,^36^,^37^. Another more recent approach involves microballoon mixing, based on reciprocating liquid between a mixing chamber and a microballoon by changing the spin rate of the disk^38^,^39^. Microballoon mixing operates at a low spin rate and low acceleration compared to the other mixing approaches and occupies no additional space on the disk^40^. This is an advantageous feature as it reduces the cost for fabrication of portable spinning system to a considerable extent.

In this paper, we describe a combination of cross-linked polymethacrylate microspheres, acting as spherical bioreceptor surfaces, with a microfluidic disk for sensitive and selective dengue virus (DV) detection. Widespread in tropical and subtropical regions, dengue fever (DF) became one of the most fatal infections that can be a worldwide threat by travelers who contracted the infection^41^,^42^,^43^. This viral disease initiates with a fever (DF) but can further develop into more severe manifestations such as dengue hemorrhagic fever (DHF) and dengue shock syndrome (DSS)^41^,^43^,^44^,^45^,^46^. With annually 400 million infections and 21,000 deaths, early detection of DV remains as a major issue^47^. In this study, an effective hybridization of two polymeric platforms is proposed for highly sensitive and selective detection of DV. We have incorporated synthetic polymethacrylate microspheres with tailored surface chemistry and controlled diameter range into a microfluidic disk equipped with microballoon mixing system as shown in Fig. 1a. Microfabricated platform, in this study, accelerates the loading/extraction procedure while applied microspheres are functionally engineered to involve all the major forces in biomolecular interaction. Combination of the functionalized microspheres with microfluidic platform benefited from mixing system is expected to enhance the detection signal and offer significant sensitivity and selectivity improvement in comparison to the conventionally available assays. A well-established protocol of sandwich enzyme-linked immunosorbent assay (ELISA) has been used for DV detection (Fig. 1b). In response to the rotational frequency changes of the microfluidic disk, the analyte solution reciprocates between microballoon and mixing chamber. Such phenomenon, in principle, provides better accessibility between biomolecules and microspheres, which subsequently results in higher chance of DV detection. The outlines of the described research are as follows: (i) performance comparison between microspheres in 96-well plates and microfluidic disks in contrast to the conventional method; (ii) optimization of the microspheres’ dose for integration into the microfluidic disk; (iii) incubation time reduction by mixing; and (iv) evaluation of the assay regarding surface chemistry and available functionalities (Fig. 1c). In that path, important parameters such as sensitivity, specificity, accuracy of the developed methodology along with limits of detection were thoroughly discussed.

## Methods

### Cross-linked methacrylate microspheres

**Figure 1:**
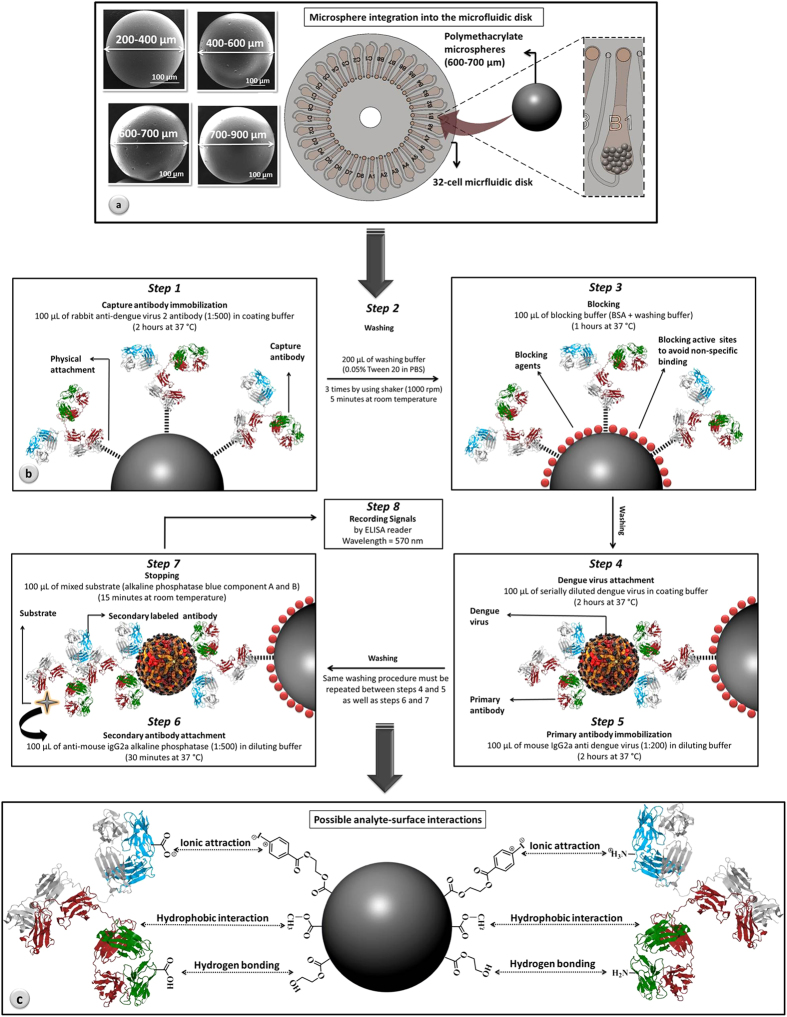
Overall interpretation of the procedure: (a) integration of the optimized microspheres into the microfluidic disk equipped with micromixing system; (b) detailed illustration of the sandwich ELISA aimed for DV detection; (c) different immobilizing interactions between microspheres and antibodies at the interface. Microspheres are yellow in their color but in the schematic representation, they are depicted in gray for better contrast.

Polymethacrylate microspheres were synthesized at INterface BIOmaterials (Geleen, the Netherlands) via proprietary suspension polymerization process. Different monomers including methyl methacrylate (MMA), 2-hydroxylethylmethacrylate (HEMA), 4-iodo-benzyloxo-ethyl methacrylate (4-IEMA), and tetraethyleneglycol dimethacrylate (TEGDMA) were cross-linked to shape microspheres in the form of polymethacrylate networks (Scheme 1S). Presented micro-scaled spheres, have been well characterized, prior to application, in terms of storage stability, hemo- and cytocompatibility, structure and absence of leachable components etc^48^. Developed microspheres are slightly hydrophilic in their nature, which is an advantage for the intended use as biorecognition platforms, since they will not tend to cluster in aqueous media and pack loosely in the assay. Ploymethacrylate microspheres were size-sorted by sieving in order to categorize the spheres in the diameter ranges as follows: size 1 (200–400 μm); size 2 (400–600 μm); size 3 (600–700 μm); and size 4 (700–900 μm). Different size categories of the spheres have been examined in analytical assay, from which the optimized diameter range (600–700 μm) has been chosen for integration into the microfluidic systems (Fig.1a).

### Morphology analysis of the microspheres by scanning electron microscopy (SEM) and optical microscopy

Morphological analysis of the polymethacrylate microspheres from different size groups was performed with SEM equipped with a field emission gun (FESEM, JEOL, JSM7600F) operated at an accelerating voltage of 0.5 kV. Prior to imaging, samples were mounted on double-sided conductive carbon tapes and coated with platinum for the purpose of avoiding surface charging. Size distribution of the spheres were calculated by images resulted from optical microscopy (OLYMPUS, BX51TRF, Japan). Diameter measurements were conducted for 500±5 randomly chosen microspheres from each size category.

### Design and fabrication of microfluidic disk

**Figure 2:**
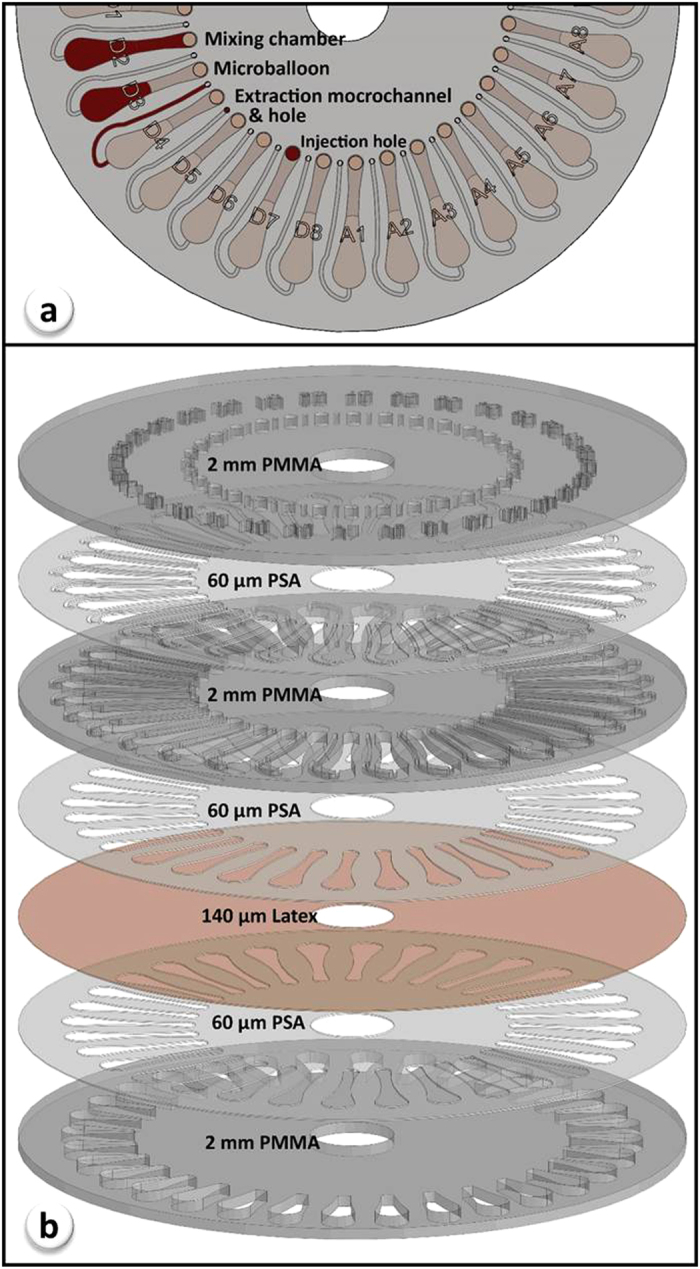
Microfluidic disk’s components: (a) microfluidic features of a mixing unit; (b) arrangement of PMMA, Latex, and PSA layers used in fabrication of microfluidic disk.

Each microfluidic disk was composed of 32 mixing units in an 8 × 4 format, and the geometry of microfluidic features were optimized based on the volume of the liquids and maximum dosage of the microspheres. Each mixing unit was comprised of injection hole, extraction hole, extraction microchannel, and mixing chamber with microballoon as shown in Fig. 2a. In order to facilitate loading of the microspheres into the microchambers and to prevent them escaping from mixing units during liquid extraction, the cross sectional area of injection holes (4.91 mm^2^) were designed significantly larger than extraction microchannels (0.28 mm^2^).

Details of the disk manufacturing and the experimental setup used in the current study were previously reported^39^. Briefly, a microfluidic disk with microballoons is composed of three poly methylmethacrylate (PMMA) disks (Asia Poly Industrial Sdn Bhd, Malaysia), one layer of latex (Accuflex Sdn Bhd, Malaysia) and three pressure sensitive adhesive (PSA) disks (FLEXcon, USA). Arrangement of PMMA, latex and PSA layers used in fabrication of the disk is demonstrated in Fig. 2b. Mixing chambers and microchannels (700 μm width × 400 μm depth) were made from cavities and engraved lines in the middle PMMA disk, respectively, using a computer numerical controlled (CNC) machine (2525 CNC Engraving & Routing, Vision, USA). The CNC machine was also used to create injection/extraction holes and labels in the top PMMA disk. A cutting plotter machine (Puma II, GCC, Taiwan) was employed to cut-out microfeatures in the PSAs, as shown in Fig. 2b. The seven layers were then aligned and stacked on top of each other and kept in a screw-compressor-clamp for an hour.

### Microballoon mixing in microfluidic disk

**Figure 3:**
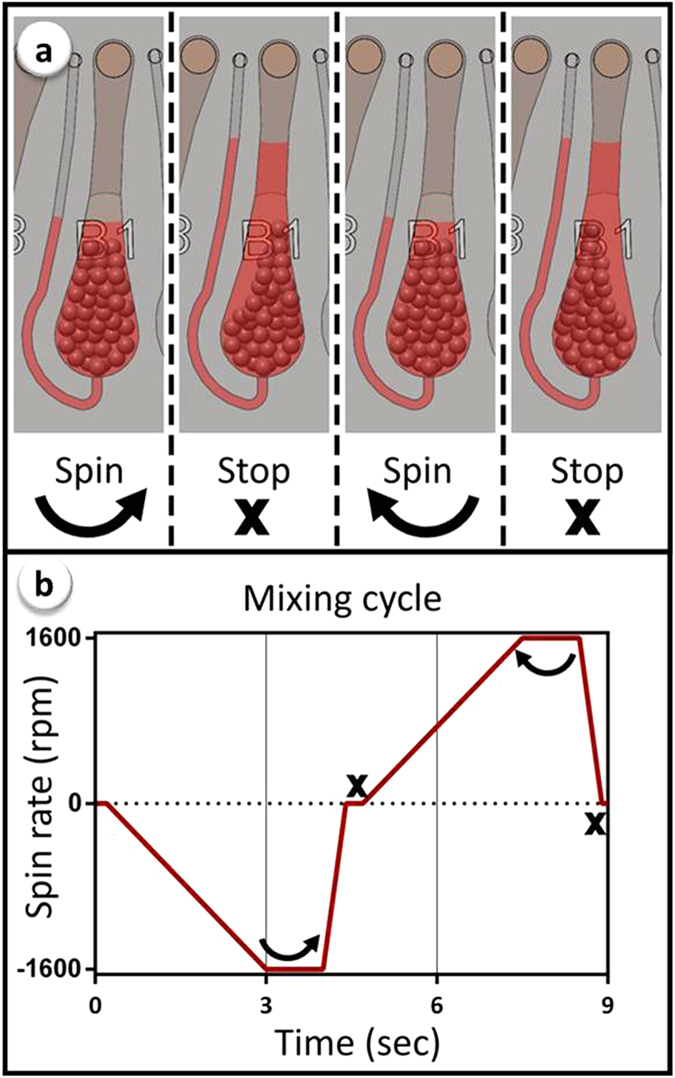
Liquid reciprocations inside a mixing chamber (a); and spin profile of the microfluidic disk during a complete mixing cycle (b). Centrifugal force at high spin rate has forced the liquid to expand the microballoon that results in a decrease in liquid level inside the mixing chamber. Afterwards, reducing the spin rate has contracted the microballoon to push back the liquid into the mixing chamber again.

Microballoon mixing is based on cyclic expansion and contraction of the latex membrane corresponding to the changes of the disk’s rotational frequency. Centrifugal pressure at high spin rate has forced liquid to accommodate inside the microballoon. Reducing the spin rate of the disk, in contrast, released the stored elastic energy of the expanded microballoon, and pushed the liquid back into the mixing chamber. Therefore frequent increase/decrease of the spin rate caused reciprocation of the liquid between microballoon and mixing chamber. Changes of the liquid level inside the mixing chamber corresponded to the amount of liquid that has been transferred into the microballoon, and were controlled by adjusting the spin rate of the disk. Details of a custom made spin control system with a high speed camera, which was used for optimizing the spin profile of mixing cycles were previously reported^39^. In order to keep all the microspheres in contact with liquid during entire mixing process, the spin profile of mixing cycles was determined based on the maximum weight of the microspheres. Therefore, a mixing unit was loaded with 80 mg of microsphere in the 600–700 μm size range, and 100 μl of red coloured liquid (selected from ELISA buffers), similar to the volume of utilized reagents in conventional assay. Spin rate of 1600 rpm, at which the liquid inside the mixing chamber almost covers the microspheres (Fig. 3a), was chosen as the optimum spin rate of microfluidic disks in the experiments. A portable spin system was used for ELISA experiments (supplementary section, Fig. 5S and Fig. 6S). Microfluidic disk was set to continuously reverse the rotation direction after the maximum speed of +1600 rpm or −1600 rpm was reached (Fig. 3b). The spin profile of the microfluidic disks during an individual mixing cycle is presented in Fig. 3b.

### Sandwich ELISA

Known as the most specific and reliable protocol, sandwich ELISA was chosen as the method of interest in order to minimize the risk of non-specific bindings and to obtain reasonably accurate results. Sandwich ELISA takes place when enveloped DV (in the present case) is locally immobilized between two analytes of choice: capture antibody (rabbit anti-dengue virus 2 antibody, ab155042, Abcam. US) and primary antibody (mouse IgG2a anti DV, ab155863, Abcam. US). The propagation of the capture and primary antibodies occurred in different hosts therefore these two biomolecules are incapable of binding to each other. A secondary labeled antibody (anti-mouse igG2a alkaline phosphatase, ab97242, Abcam US), conversely, can further bind to the primary antibody and generate detection signal (Fig. 1b). Resultant signal intensity can be correspondingly enlarged, with the concentration of target analyte. The data sheets of the purchased commercial products state that the sandwich ELISA exhibits minimal chance of cross-reactivity or interface with other analytes (less than 2%). Pre-determined dosages of the microspheres were placed inside the ELISA 96-well plate and microfluidic chamber and sandwich ELISA was performed in both. A conventional ELISA was also conducted as a control along with every set of the experiment. In order to minimize experimental variability, all immunoassays were performed under the exact same condition and by using the same reagents. All the datasets obtained in this study were subjected to the standard calibration curve analysis. No significant intraday variability was observed for the conducted assays. To determine the optimal dosage of the spheres for the best detection performance, different loadings of the spheres (10 mg, 20 mg, 40 mg and 80 mg) were integrated into the 96-well plates and microfluidic disk. Nonetheless, experiments pertaining to the detection range, calibration curves and comparison studies were all conducted with only 20 mg of spheres (justification of this choice can be found further in the text). The enveloped dengue virus used in this study was prepared via a clinical isolation of dengue serotype 2 from a patient’s serum sample (DV2-isolate Malaysia M2, Gen Bank Toxonomy No.: 11062). A detail explanation of virus propagation and isolation procedures can be found in supplementary section. Moreover, a detailed description of sandwich ELISA is also provided in supplementary section. Depending on the purpose of the conducted assay, variety of virus concentrations was prepared through serial dilution. For instance, to calibrate the assay, 4 serial diluted virus solutions in the concentration range between 3.5 × 10^−2^ p.f.u/mL to 3.5 × 10^−6^ p.f.u/mL were used. Calibration curves have been plotted by conversion of the data to the logarithm values. Detection range was investigated by running the assay in the concentration range of 3.5 × 10^6^ p.f.u/mL to 3.5 × 10^−6^ p.f.u/mL. The comparison of the detection signal generated from well plate and microfluidic disk as well as from controls was performed with a selected virus concentration of 3.5 × 10^2^ p.f.u/mL. Negative controls were calculated from the assays that were conducted in the absence of virus (n = 16). Cut-off values for each individual system were considered as twice of the mean values resultant from the negative controls^44^. Only those samples, which have resulted in optical density (OD) higher than calculated cut-off values were interpreted as positives. All of the detection results, which are accompanied by their negative controls, have been plotted after subtracting cut-off values from the original data. Based on the performance of the microspheres (taken from different size categories) the conducted assay in well plates has been thoroughly evaluated and results are presented in supplementary section, Table 1S. Furthermore, the selected size of the spheres, integrated inside the microfluidic disk, has also been evaluated and compared with the performance of the same size spheres in the well plate and conventional assay. A total of 162 replicates including 114 positive and 48 negative samples were examined in sandwich ELISA to investigate the sensitivity and specificity of the proposed methods in comparison to the conventional clinical practice, ELISA. Furthermore, accuracy of the assay was calculated by true and false negative/positive readings in contrast to the total number of replicates^49^. Limit of detection (LoD) for each individual system was determined from the average standard deviations (for the minimum DV concentration) and slopes of the calibration curves^50^.

## Results

### Morphology, size distribution and surface area analysis of the polymethacrylate microspheres

**Figure 4:**
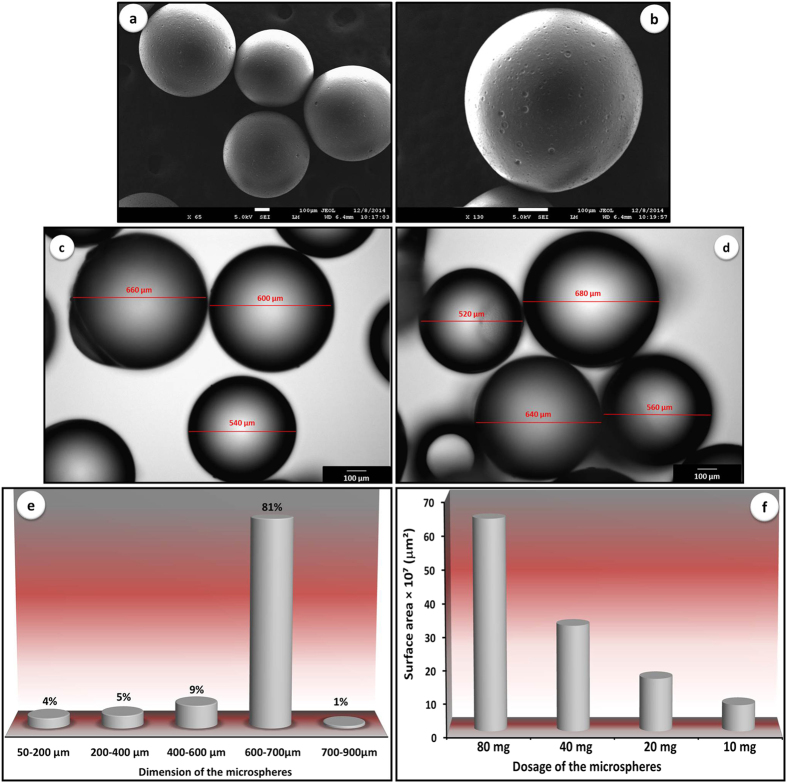
Microspheres analysis (size 3 only): (a,b) morphology analysis by SEM; (c,d) optical microscopy images of microspheres used for size distribution analysis; (e) determined size distribution of the microspheres; and (f) calculated specific surface area per different dosages of the microspheres.

Microspheres have been thoroughly analyzed in respect to their surface morphology in SEM and representative images are shown in Fig. 4 (a,b, only images for size 3 spheres) and supplementary section, Fig. 1S (images for all size categories). The micro-scaled spheres featured perfectly round shapes with very smooth surfaces. The dimensions of the microspheres were found to be consistent with their expected size ranges obtained from sieving procedure. Consistently smooth surface morphology enables discussion to be focused on the effect of size domain and consequently specific surface area, on immobilization efficiency. Furthermore, immobilization behavior and detection performance can be studied as a function of surface chemistry since the analysis has shown uniform surface morphologies for all investigated microspheres (supplementary section, Fig. 1S).

Representative optical images of the selected microspheres (size 3) are presented in Fig. 4 (c,d). Figure 4e displays size distribution, calculated from optical images of the spheres. As can be observed, 19% of the size 3 spheres were recorded to fall outside of the predicted size range (600–700 μm). Nonetheless, the fact that 81% of the microspheres are within the expected diameter range validates further discussion based on the size distribution. A detailed comparison of size distribution for all diameter categories is provided in supplementary section (Fig. 2S).

Available specific surface areas of the microspheres can be precisely calculated from a detailed size distribution analysis. As expected, surface area increases with increased unit mass of the spheres (Fig. 4f). Therefore lowest and highest specific surface areas were measured for 10 mg and 80 mg of the microspheres, respectively. Higher specific surface area, in principle, provides better accessibility of the macromolecules to the bioreceptor’s surface hence causing higher probability of analyte-surface binding. Figure 3S of the supplementary section represents specific surface areas (μm^2^) of each size groups per varied dosages of the spheres (mg). Since the discussion is concentrated on size 3 microspheres, only calculated specific surface area for this particular size is depicted in the text as Fig. 4f.

### Detection range study of the microspheres from different size categories inside the well plate

**Figure 5:**
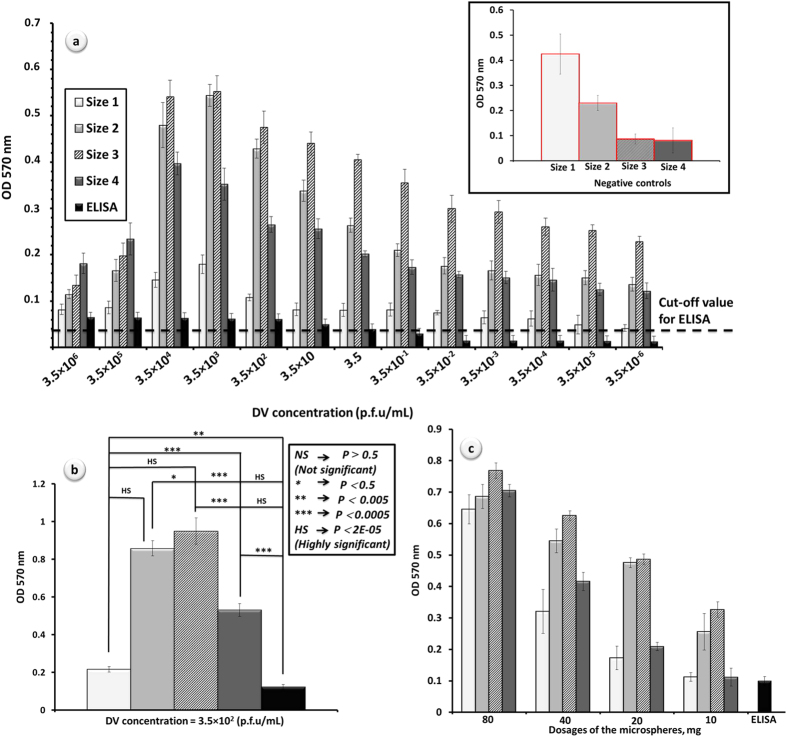
Performance of the microspheres in DV detection: (a) detection range study performed on the microspheres (20 mg) of different sizes and conventional ELISA (polystyrene) in a broad range of DV concentration; (inset) representative negative controls in sandwich ELISA at which the assay was conducted in the absence of DV (spheres’ dosage = 20 mg); (b) statistical analysis of the performance of the spheres from different size groups (c) dosage influence on detection performance conducted on the spheres of different size ranges via sandwich ELISA (DV concentration = 3.5 × 10^2^ p.f.u/mL). Negative controls for parts b and c of this Figure follow the insert from part a.

Pre-determined dosages of the microspheres (different size categories) were placed inside the ELISA 96-well plate (supplementary Fig. 4Sa–4Sb), for which sandwich ELISA has been performed. Variety of virus concentrations has been used in order to investigate detection range of the developed platforms in comparison to commercial assay. Performance of the microspheres from different sizes was studied and compared in this broadened range of DV concentrations and results are plotted in Fig. 5a. Regardless of the applied DV concentration, microspheres of the smallest dimension (size 1) have yielded in the lowest performance level among all. Smallest size in our micro-scaled spherical platforms might attract substantial future attention in rapidly developing technology that has a tendency to present products of micro- and preferably nano-scales^51^. From the practical point of view, however, conducted clinical assay with microspheres size 1 encountered major difficulties. For instance, since the same dosage of size 1 contains more spheres than other sizes, expectedly, this category generates higher experimental error as a result of incomplete washing procedure. Furthermore, considerable loss of size 1 spheres in the routine pipetting was unavoidable, which, in turn, limits the efficiency of this particular size category in the assay. Microspheres of the smallest size would enter or block the pipette tips even when the smallest tip size has been chosen. Mentioned factors can have a considerable contribution in large errors of the assay, which has been conducted by using this size category (Fig. 5a, inset). Described experimental drawbacks have not been observed when the assay was performed with other size groups of the microspheres. In particular, microspheres have a density of approximately 1.2 g/ml so they readily sink in aqueous media. This is an advantageous feature that facilitates pipetting of supernatants while microspheres (quantitatively) stay in the well.

As it can be seen from Fig. 5a, detection signal intensity initially increased as DV concentrations decreased. This phenomenon has most likely occurred as a result of large size of the biomolecules used in the assay as such macromolecules can lose their activity by denaturation caused by steric repulsion^51^. Therefore, lower concentrations of such biomolecules facilitate analyte-surface interaction in a more efficient manner. This increase in detection signal, however, was limited to the certain threshold (DV concentration = 3.5 × 10^3^ p.f.u/mL), from which detection signal has gradually dropped to the lowest examined concentration levels. Nevertheless, presented results illustrate that developed platforms are adequate for their use in diagnostics, as the detection signal remained positive even in the lowest concentrations of DV (3.5 × 10^−6^ p.f.u/mL). It should be noted that in all of the DV concentrations, conventional ELISA yielded in relatively poor and uncertain levels of detection in comparison to the assay performed with polymethacrylate microspheres. The statistical analysis presented in Fig. 5b, shows greater performances of the microspheres in comparison to ELISA. From presented results it can be observed that size 3 microspheres showed highly significant performance in comparison to the rest (Fig. 5b). Although microspheres of size 1 have the highest specific surface area (supplementary section, Fig. 3S), the experimental error introduced by the loss in pipetting process has an obvious impact on DV detection (Fig. 5a). Another factor that contributes to such a poor performance is the significant negative control recorded for size 1 spheres. In fact, both sizes 1 and 2 have shown intensive signals from respective negative controls, which affect their performance in comparison to other sizes (Fig. 5a, inset). Furthermore, size 4 microspheres are the easiest to handle manually, however, their performance is lower than size 3 due to the difference in specific surface area (supplementary section, Fig. 3S). It could be concluded that the size 3 microspheres has the optimal dimension for reliable and robust detection of DV in ELISA experiment and for subsequent integration into the microfluidic disk.

### The influence of dosage on the detection performance of the microspheres from different size categories

Dosage influence on detection performance was investigated in respect to optimization of the methodology and results are depicted in Fig. 5c. Relatively higher performance of size 3 microspheres in all of the different dosages can be clearly observed from the presented results. As it could be predicted, the signal intensity decreases with the loading of the microspheres. This observation proves that available active sites for protein attachment are the function of the spheres’ dosage involved in bimolecular interaction. For reminder, based on the obtained results from previous section, size 3 spheres (600–700 μm) have been chosen as the optimum group for integration into the microfluidic disk. Negative results of different loadings of the spheres inside the well plate have shown minor difference (supplementary section, Fig. 7S). Therefore, arbitrary negative controls for 20 mg of the spheres from different size categories are depicted as representatives in Fig. 5a (inset).

### Detection range study on the selected microsphere category (size 3) integrated into the microfluidic disk and the influence of microspheres’ dosage on detection performance of microfluidic disk

**Figure 6:**
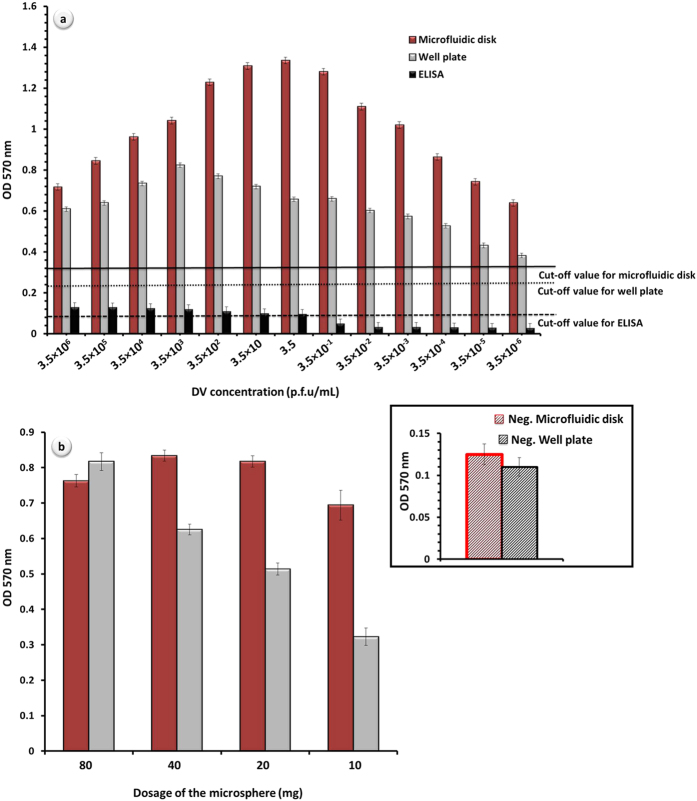
Performance of the microspheres in DV detection: (a) detection range analysis for the well plates and microfluidic disks with microspheres (20 mg of size 3 spheres) in comparison to the conventional ELISA (depicted results are original data along with their cut-off values); (b) influence of spheres’ dosage (size 3 spheres) on the detection performance (DV concentration = 3.5 × 10^2^ p.f.u/mL, results are plotted after subtraction of the cut-off values); (inset) negative controls for microspheres integrated well plate and microfluidic disk (spheres’ dosage = 20 mg).

Optimized size category of the microspheres has been integrated into the microfluidic disk as shown in Fig. 4Sc, supplementary section. Same virus concentrations (as for well plate) were used to investigate the detection range that can be obtained from microfluidic disk in contrast to that achievable in a traditional commercial assay. Figure 6a represents the performance comparison between spheres integrated well plate and microfluidic disk over a broad range of DV concentration. Figure 6a contains plotted original data along with their cut-off values. Obtained signal intensities from either method are in accordance with previously reported OD values for DV detection^43^,^44^,^46^. A simple visual observation immediately emerges that micromixing of the spheres in microfluidic disk, has remarkably enhanced the detection signal (approximately 10-fold higher than conventional ELISA and 2-fold greater than well plate with microspheres, Fig. 6a, DV concentration = 3.5 p.f.u/mL). Following the same trend as before, detection signal in both cases (well plate and microfluidic disk) increases as DV concentration decreases. Such increasing trend in detection signal, again, has changed the direction towards decreasing OD values from DV concentration = 3.5 p.f.u/mL. It is important to note that the turning point in current case (microfluidic disk) was shifted to the considerably lower DV concentration in comparison to the previously discussed data depicted in Fig.5a (well plate with microspheres). Although OD values eventually dropped, the results presented here clearly illustrate that detection signal remains positive even in the lowest examined concentration levels (3.5 × 10^−5^ and 3.5 × 10^−6^ p.f.u/mL) that are not possible to be detected with a conventional ELISA.

Different loadings of the microspheres were utilized in microfluidic disk in the same fashion as were used in well plate. Negative results for different loadings of the spheres inside the microfluidic disk have shown no major difference (supplementary section, Fig. 8S). For that reason, obtained negative controls for 20 mg of the loaded spheres with 30 minutes incubation time are depicted as representatives in Fig. 6b (inset). As it was described, the signal intensity recorded from well plate decreases as the amount of microspheres decreased. When microspheres are integrated into the microfluidic disk, however, detection behavior does not follow the same order. Although detected signals from the microfluidic disk were significantly higher than the signal intensities from the well plate, surprisingly the highest dose of spheres has resulted in lower signal intensities in this case (80 mg < 40 mg ≥ 20 mg). Micromixing system designed for the microfluidic disk provides higher reaction rate by imposing better chance of connectivity between biomolecules and microspheres. In the case of higher dosage of the spheres (80 mg), lack of spatial freedom has resulted in less chance for bioreceptors to interact with the target proteins. Detection signal intensity increased by integration of lower doses of the spheres (40 mg and 20 mg) into the microfluidic disk as a result of optimal micromixing. Eventually, the detection signal on the microfluidic disk slightly dropped when using only 10 mg of spheres. For obvious reasons, 20 mg of microspheres was selected as an optimum load for integration into the microfluidic disk. Nevertheless, it is of a great importance to know that even the highest dosage of the microspheres (80 mg) would still be favorable for integration into the microfluidic devices of specific designs. Figure 6b has been plotted after subtraction of the cut-off values. However, the negative controls have been added to the Fig.6b for the ease of discussion. Although generated results from negative controls have been slightly higher than conventional assay, both methods were observed to be reproducible to a high degree.

### The effect of micromixing on incubation time

**Figure 7:**
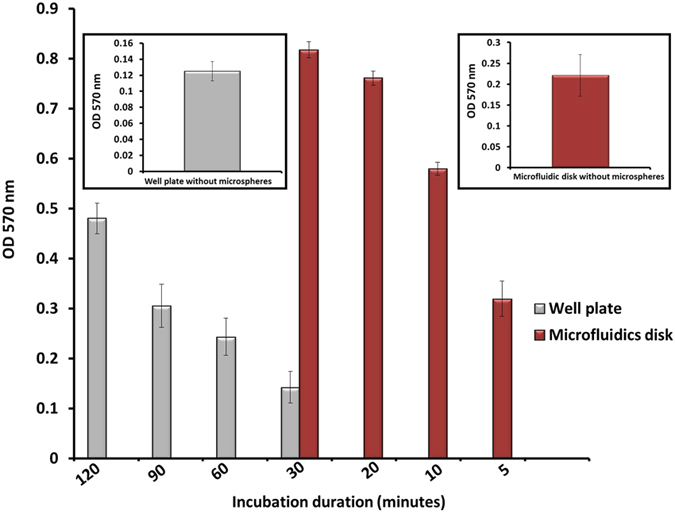
Comparison of the detection performance over different incubation periods (dosage of the spheres = 20 mg and DV concentration = 3.5 × 10^2^ p.f.u/mL); insets depict blank controls, which are the detection signals generated from the well plate and microfluidic disk without microspheres (negative controls are provided in Supplementary section, Fig. 9S).

The influence of micromixing on incubation time was investigated and results are presented in Fig. 7. As blank controls, sandwich ELISA has been performed inside the 96-well plate as well as microfluidic disk without microspheres, operating at the incubation time of 120 minutes and 30 minutes, respectively (Fig. 7, insets). Negative controls of the assay conducted in different incubation times can be found in supplementary section (Fig. 9S). Integration of the micro-scaled spheres into the microfluidic disk and subsequent application of micromixing has resulted in a substantial time reduction from regular incubation duration of 120 minutes to minimum of 5 minutes. Such a significant reduction (24-fold less time consumption) occurred while preserving the positive detection signal. Applied mixing mechanism enables the analyte solutions to frequently travel back –and–forth across the microfluidic chamber thus makes an improved contact between biomolecules and available active sites of the bioreceptors. Microspheres had the partial mobility in the chamber as well, which further enhances the reaction statistics. Tedious and lengthy assay of numerous incubation steps can be rapidly performed with such a significant time reduction, yet recorded OD values proved a notable detection enhancement in comparison to the conventional method, ELISA.

### Evaluation of the assay

Different DV concentrations have been chosen in the range of 3.5 × 10^−6^ p.f.u/mL to 3.5 × 10^−2^ p.f.u/mL for constructing calibration curves for different size categories of the spheres, from which size 3 spheres were chosen for integration into the microfluidic disk. Plotted calibration curves and carefully evaluated analytical parameters for each individual size groups can be found in supplementary section (Fig. 10S and Table 1S). Same DV concentrations have been used to calibrate microsphere integrated microfluidic disks as well (supplementary section, Fig. 11S). Calibration data have shown that the developed methods have higher level of precision than routine analytical assay, ELISA.

When the assay is performed in the absence of virus, highly accurate analytical systems are expected to generate no positive detection signal, as the biomolecular sequence has intentionally been broken. Non-specific bindings at deliberate negative and positive replicates can be quantitatively compared to the actual negative and positive results and establish the sensitivity and specificity of the methodology^49^. This special analysis illustrates the possibility of the errors in the assay thus provides useful information regarding reliability and accuracy of the developed analytical system. A total of 38 positive and 16 negative controls from an overall of 54 replicates were examined to assess the performance of each individual system. Table 1 represents evaluation parameters for the assays conducted in well plate and microfluidic disk (20 mg of size 3 spheres) with the incubation time of 120 minutes and 30 minutes, respectively. Negligible difference in performance of the assay was observed as a function of the incubation time. Therefore, presented negative results (true and/or false) are chosen from 30 minutes of incubation, as representative. Developed methods, in general, have drawn a great deal of sensitivity in comparison to the conventional assay. Proposed microspheres integrated systems, in this study, exhibited an equivalent level of sensitivity (97.36%), which was considerably improved in contrast to clinical method (76.31%). Specificity of the proposed methods has also found to be in a greater range than clinical assay (Table 1). Further data analysis demonstrated a considerably higher level of accuracy for the microfluidic disk and the well plate compared to the conventional ELISA (77.77%). Table 1 also represents the calculated LoD values for the two developed methodologies. Unlike conventional ELISA, proposed systems have proven to be capable of detecting only a few plaque forming units of DV in blood serum. In contrast, conventional ELISA was only capable of detecting a minimum concentration of 5 × 10^3^ p.f.u/mL, which is categorized as relatively late stages of DF (day 4–6 of the fever)^44^,^52^,^53^,^54^. This finding is of crucial importance as the major concern for early detection of DV can be addressed in future optimization and development of proposed methodology.

Despite existence of several different techniques for regeneration of the developed system such as treatment with strong oxidizer, acid/base components and/or concentrated salts^55^,^56^, reusability of the proposed methodology is no subject of the debate. Microfluidic disks have been fabricated as a disposable platform that, in the industrialized level, can be purchased in a reasonable price and are designed for their special application in remote areas (extreme point of care, EPOC)^55^. The fact that a single microfluidic disk can simultaneously be applied for detection of several types of analytes (multiplexing) endorses such devices as cost-effective diagnostic systems and justifies the disposability of the micro-fabricated platforms.

### The impact of surface chemistry on DV detection

The excellent performance of the microspheres inside the microfluidic disk can be largely ascribed to improved accessibility of the biomolecules to the specific surface areas of suspended spheres, which was facilitated by micromixing. Apart from the considerable specific surface area, other strong reasons laid the underpinning of such significant performance that necessitates further discussion. As it was defined in the methodology section (microsphere preparation), different monomers participated in polymerization reaction in order to shape the final product of polymethacrylate microspheres. According to its individual molecular structures, each monomer leaves its special chemical finger-print behind, on the microspheres’ surface in the form of different functionalities such as –OH groups, methyl ester groups (–COOCH_3_), aromatic groups, iodine (bound to an aromatic group) and short ethylene glycol chains (Fig. 1c). Correspondingly, such a multitude molecular structure of the spheres promotes binding with target biomolecules via a plethora of direct interactions. Among various forces that impact biomolecular interaction, three major forces are known to be the most influential parameters in analyte-surface interaction. Namely, ionic attraction, hydrophobic interaction and hydrogen (H)-bonding, have frequently been reported and discussed for their significant impact on immobilization^52^,^53^,^57^. Ionic attraction is relatively weak compared to the other forces and can be often considered negligible while H-bonding has proven to be the strongest force for the most substantial protein attachment^57^. In the present case, polymethacrylate microspheres provide the opportunity for all the mentioned forces to play the role in biomolecular interaction. Figure 1c suggests possible interactions that might occur between biomolecules and microspheres. Aromatic/iodine groups of the spheres may impart ionic attraction (electrostatic interaction) with –NH_3_^+^ and –COO^−^ groups of the proteins in their amphoteric form. Relatively hydrophobic groups on the microspheres’ surface (–COOCH_3_) might, in its own way, promote the well-known force of hydrophobic interaction in attracting proteins to the surface^57^. In a completely different manner, available –OH groups of the spheres might interact with both –COOH and –NH_2_ groups of the biomolecules via H-bonding.

When development of biosensor with warrantied shelf-life and substantial performance is in the center of attention, stability of the surface functionalities makes a stronger sense. Modified surfaces of the biosensors often lose their activity over time due to the reorientation of the surface functionalities. Generated surface functional groups often tend to reorganize themselves to occupy lower levels of energy, thus result in deactivation of the modified platforms. Since available functional groups on the surface of the polymethacrylate microspheres have been formed through the synthesis reaction, they are part of the chemical history of the polymer system, hence would not be affected by aging or other similar phenomena^52^. It is also of a great importance to know that both developed platforms, microspheres and microfluidic disk, are plastic materials thus highly stable in the integrated form.

## Discussion

In summary, synthetic polymethacrylate microspheres with specifically designed surface chemistry and morphology have been integrated into the microfluidic disk. Developed micro-fabricated platform in this study, was equipped with an efficient micromixing system that resulted in a higher chance of analyte-surface interaction and subsequently enhanced DV detection. Detection performance of the microspheres inside the microfluidic disk was compared with the obtained results from conducted assay with microspheres inside the 96-well plates and conventional ELISA (as a reference). With approximately 10-fold greater detection signal than conventional ELISA and 2-fold higher signal than integrated microspheres into the well plate, proposed microfluidic system has drawn a great deal of performance in DV detection. Developed methodology has reduced the incubation time of the assay from 120 minutes (routine procedure) to minimum of 5 minutes while preserving the strong positive detection signal. Such a significant curtail in time consumption is essential as the laborious and lengthy ELISA can be rapidly conducted. When a fatal disease such as dengue with the period of 7 days is the subject of diagnosis, a significant reduction in routine incubation times, which can vary from minimum 2 hours up to 12 hours, can improve the current clinical practice. According to world health organization (WHO), in the first quarter of the year 2014 alone, the outbreak of DF in Malaysia exceeds to 27,500 individual cases, which subsequently resulted in 64 deaths^58^. In such circumstances, providing timely information can serve the society with better surveillance and improved “point of care”.

Moreover, integrated microspheres into the microfluidic disk have remarkably detected only few units of enveloped dengue virus in serum (1.9 p.f.u/mL), which is highly encouraging for future fabrication of sensitive and selective diagnostic devices capable of high throughput early detection. Developed system in this study is disposable and can be industrialized in a very cost-effective manner. Based on the estimations, dengue is one of the major public health problems in 112 countries^59^. Considering the fact that given information by related organizations contains infected cases from the cities as well as rural areas, development of low cost and portable devices is necessary to reduce the burden caused by dengue, to a large extent. Detection of dengue in the early stages of infection is expected to reduce the mortality rates from 20% to below 1%^49^,^60^,^61^. Microsphere associated micro-fabricated disk, as proposed in this study, provides various opportunities for future development of portable analytical devices that can offer a simple, cost-effective and rapid diagnosis in remote areas.

## Additional Information

**How to cite this article**: Hosseini, S. *et al.* Microsphere integrated microfluidic disk: synergy of two techniques for rapid and ultrasensitive dengue detection. *Sci. Rep.* **5**, 16485; doi: 10.1038/srep16485 (2015).

## Supplementary Material

Supplementary Information

## Figures and Tables

**Table 1:** Break down values for sensitivity, specificity, accuracy and limit of detection (LoD) for microspheres integrated microfluidic disk and 96-well plate in comparison to conventional ELISA.

Platform	Microfluidic disk		Well plate		ELISA	
DV status	+	−	+	−	+	−
Positive	37	2	37	1	29	3
Negative	1	14	1	15	9	13
Total	38	16	38	16	38	16
**Sensitivity (%)**	97.36		97.36		76.31	
**Specificity (%)**	87.5		93.75		81.25	
**Accuracy (%)**	94.44		96.29		77.77	
**LoD (p.f.u/mL)**	1.9		5.12		5 × 10^3^	
