# Supplementary material for: Microsphere integrated microfluidic disk: synergy of two techniques for rapid and ultrasensitive dengue detection
Source: Sci Rep. 2015 Nov 9;5:16485. doi: 10.1038/srep16485 (PMC4637926; doi:10.1038/srep16485)
Supplement: Supplementary Information [file srep16485-s1.pdf]

Supplementary Section

Microsphere integrated microfluidic disk: synergy of two techniques for rapid and ultrasensitive dengue detection

Samira Hosseini[1,2], Mohammad M. Aeinehvand[1,2], Shah M. Uddin[1,2], Abderazak Benzina[3], Hussin A. Rothan[4], Rohana Yusof[4], Leo H. Koole[1,2,3], Marc J. Madou[1,2,5,6], Ivan Djordjevic[1,2], and Fatimah Ibrahim[1,2]

[1]Department of Biomedical Engineering, Faculty of Engineering, University of Malaya, Kuala Lumpur, 50603, Malaysia, [2]Center for Innovation in Medical Engineering, Faculty of Engineering, University of Malaya, Kuala Lumpur, 50603, Malaysia, [3]Faculty of Health, Medicine and Life Sciences, Maastricht University, the Netherlands, [4]Department of Molecular Medicine, Faculty of Medicine, University of Malaya, 50603, Kuala Lumpur, Malaysia, [5]Department of Biomedical Engineering, University of California, Irvine, 92697, United States, [6]Department of Mechanical and Aerospace Engineering, University of California, Irvine, 92697, United States

Macromolecular structure of polymethacrylate microspheres

Microspheres were polymerized in suspension polymerization by using HEMA, MMA and IBEM as monomers that have been polymerized by TEGDMA as cross linker. Chemical structure of the developed microspheres is presented in Scheme 1S.

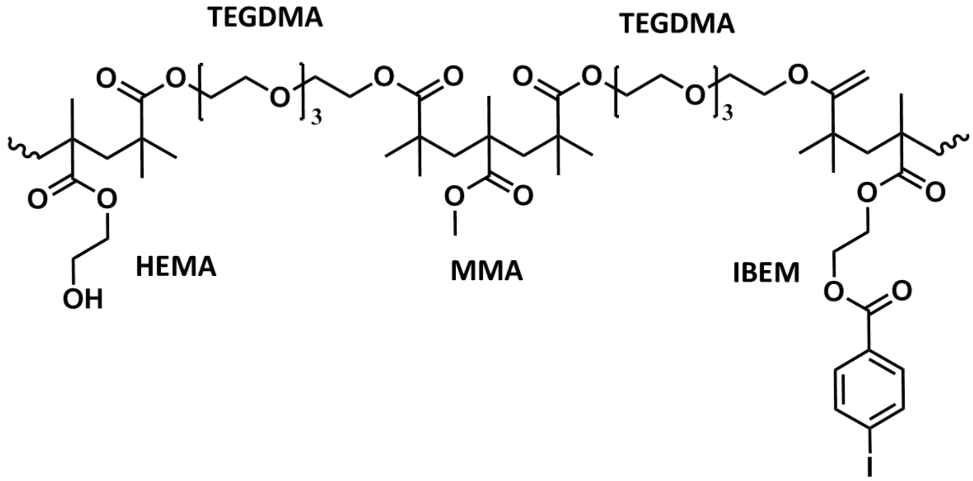

Scheme 1S. Macromolecular structure of polymethacrylate microspheres produced from HEMA, MMA and IBEM monomers cross-linked with TEGDMA.

Morphology analysis of the polymethacrylate microspheres

Surface morphology of the microspheres was recorded with SEM and selected images are presented in Fig. 1S. Microspheres, in general, revealed perfectly round shapes with smooth and uniform surfaces. SEM analysis has also shown that microspheres of different diameters have been produced in optimized distribution ranges. Figure1S (a-d) presents the images of microspheres size 1 (200-400 μm); size 2 (400-600 μm); size 3 (600-700 μm); and size 4 (700-

900 μm), respectively. Diameters detected by SEM are, to a large extent, consistent with expected sieved size ranges.

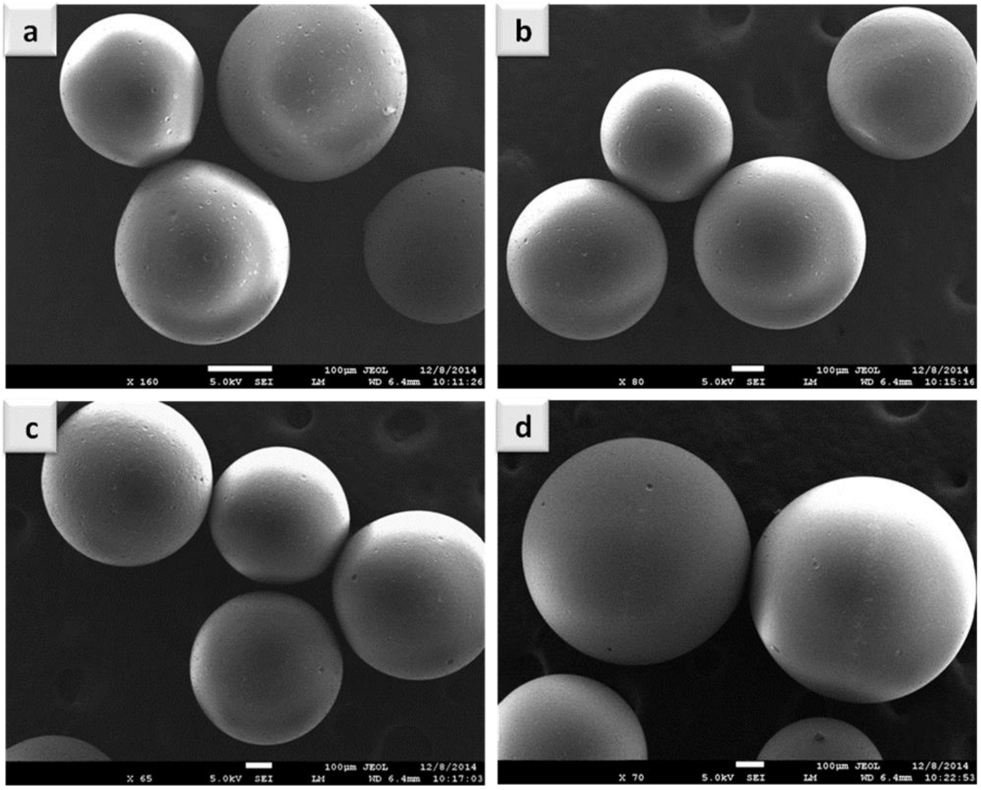

Figure 1S. Morphology analysis of microspheres by SEM: (a) size 1 (200-400 μm); (b) size 2 (400-600 μm); (c) size 3 (600-700 μm); and (d) size 4 (700-900 μm).

Size distribution analysis of polymethacrylate microspheres

Size distribution analysis of the developed microspheres for all of the size categories has been performed in order to evaluate the separation procedure. Figure 2S (a-d) displays size distribution of the microspheres, measured for 500 ± 5 spheres from the images taken by optical microscopy. Microspheres size 1 and size 2 contain (by number) 88% of the microspheres in the expected sieved size (diameter) (Fig. 2Sa and 2Sb). Figure 2Sc, represents 81% of available microspheres in the predicted diameter range (size 3) while analysis for size 4 category (Fig. 2Sd) reveals only 79% of the spheres in the nominal size range.

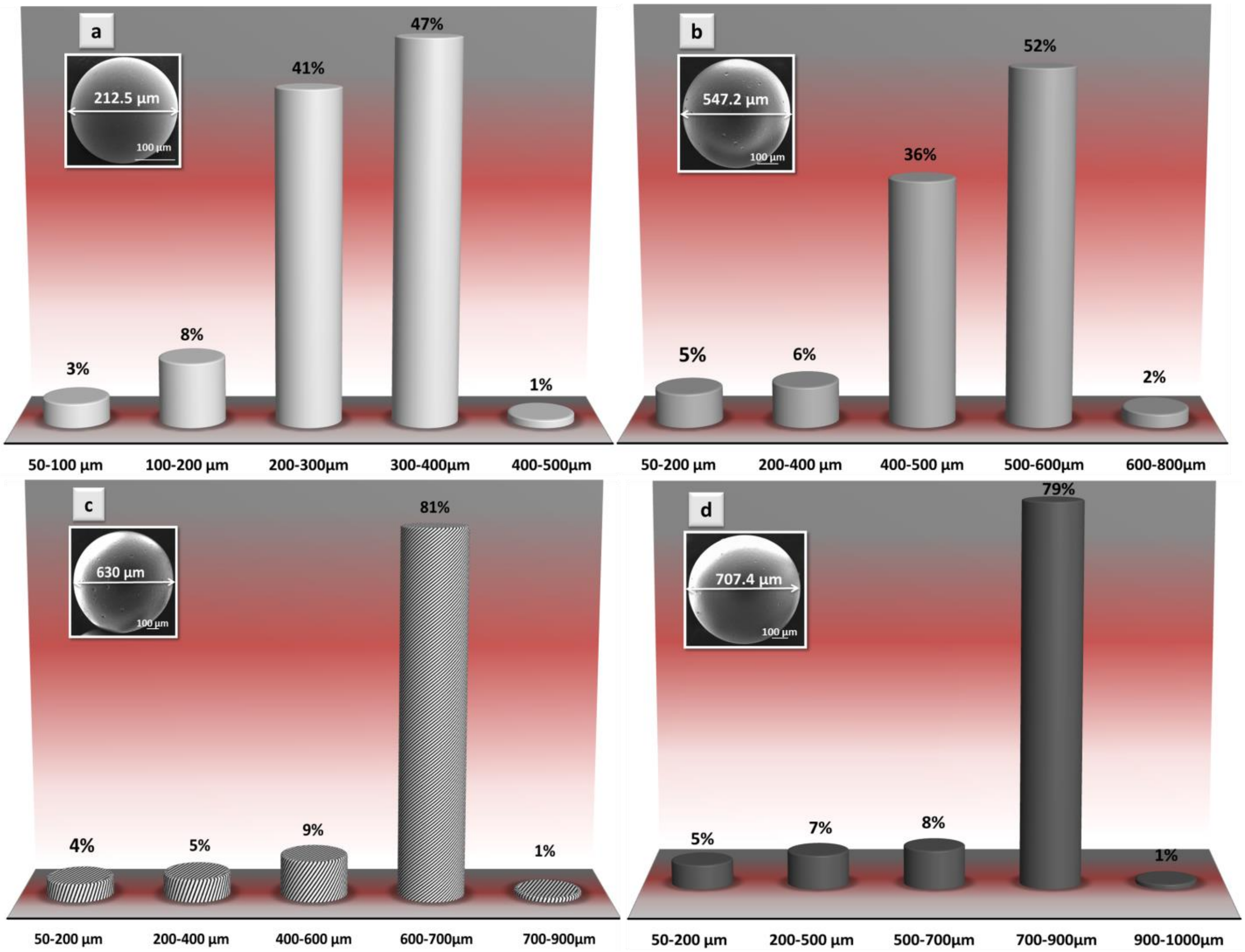

Figure 2S. Detailed analysis of the microspheres' diameter distribution in different size range: (a) size 1 (200-400 µm); (b) size 2 (400-600 µm); (c) size 3 (600-700 µm); and (d) size 4 (700-900 µm). Presented percentages in the form of graphs are the results of measurements for 500 ± 5 spheres from each size category. Insets are the representative SEM images of single microspheres from each size category along with the average diameter for that particular size group.

Surface area analysis of polymethacrylate microspheres

Detailed size distribution analysis of each size group acts as an accurate parameter for precise calculation of specific surface areas available for biomolecular interaction. Figure 3S represents a comparative study between three key elements in analyte-surface interaction: size (µm); dosage

(mg); and specific surface area (μm^2) of the spheres. Microspheres of different sizes and dosages have offered a diverse range of specific surface areas available for protein interaction (Fig.3S). It can be observed from Fig. 3S that the minimum surface area was calculated for 10 mg of size 4 microspheres.

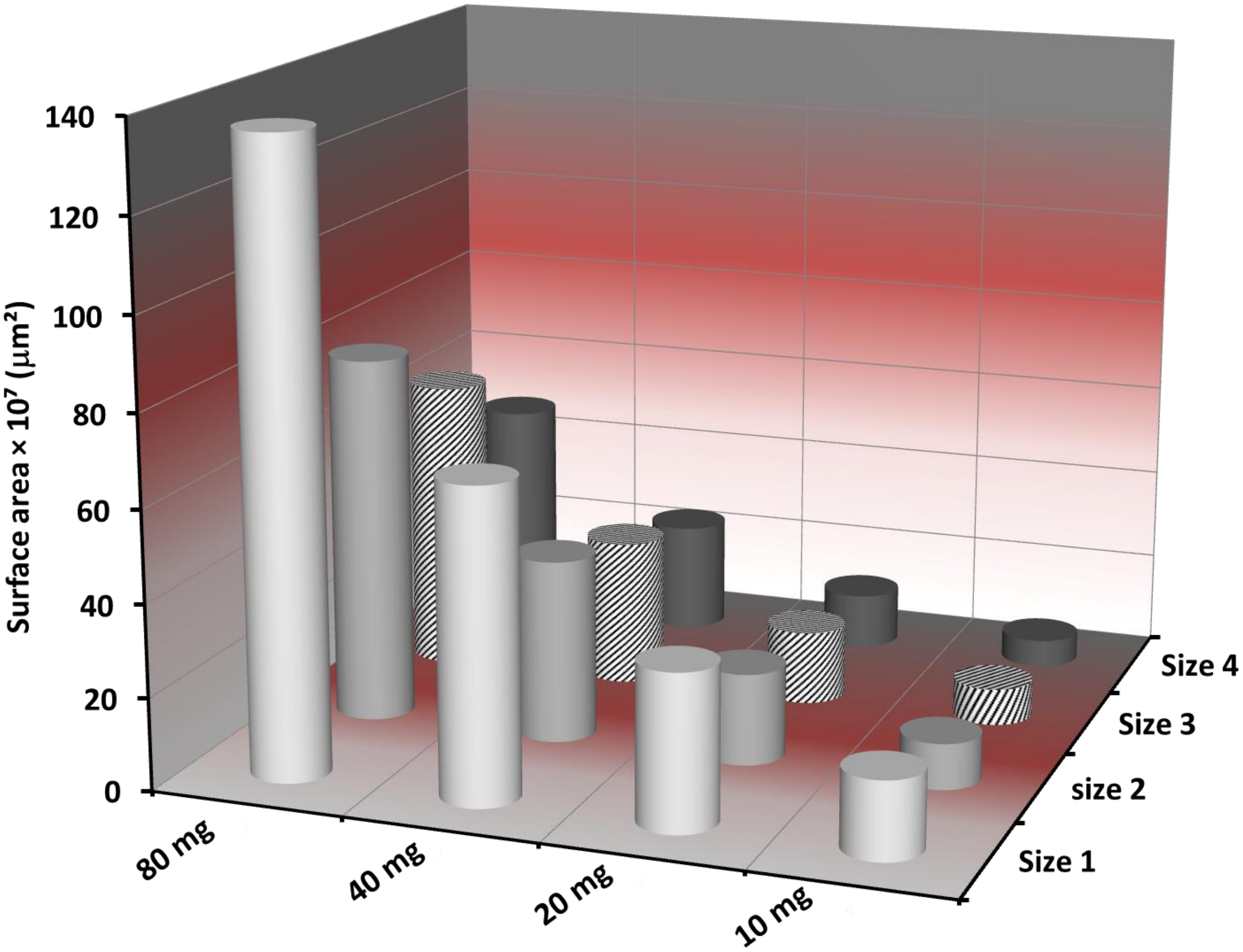

Figure 3S. Comparative study of specific surface area for different dosages of the spheres (10 mg, 20 mg, 40 mg, and 80 mg) from different size groups: size 1 (200-400 µm); size 2 (400-600 µm); size 3 (600-700 µm); and size 4 (700-900 µm).

As expected, maximum specific surface area corresponds to the highest amount (80 mg) of the smallest microspheres (size 1). Larger specific surface area allows macromolecules, in principle,

to have a higher accessibility to the surface of the bioreceptor thus creating higher probability for protein attachment.

Integration of microspheres

Selected category of the microspheres (size 3) with different dosages has been placed inside the 96-well plate as well as microfluidic disk (Fig. 4S).

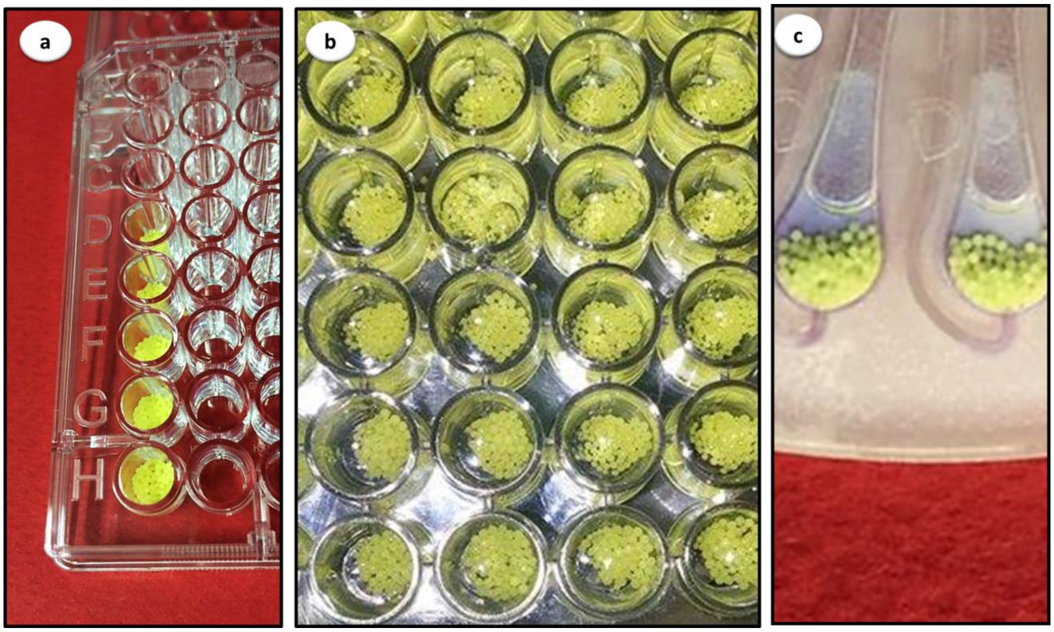

Figure 4S. Integrated microspheres into the 96-well plate (a and b) and microfluidic disk (c).

Control circuit for portable spin system

The control circuit of the spin system comprised of a microcontroller (ATmega328P-PU), a dual full-bridge driver (L298), and a conventional carbon brush direct current (DC) motor. Figure 5S shows the control circuit diagram of the spin system. The bridge A of L298 driver involves 3 microcontroller pins (PB4, PB1, and PC3) to drive the DC motor. The PB4 pin (DIRA) was used to control the motor rotational direction, the PB1 pin (BRAKEA) was used to stop motor's rotation and the PC3 pin Pulse-width modulation A (PWMA) used to control the motor's

rotational speed. The microcontroller is connected to TTL (Transistor–transistor logic) compatible bridge A inputs of L298 driver through a NAND Gate (4077D). The DC motor is connected to bridge A outputs (OUT1 and OUT2) of the L298 driver. The microcontroller and the L298 driver is powered by +5 volt (V) of DC power supply.

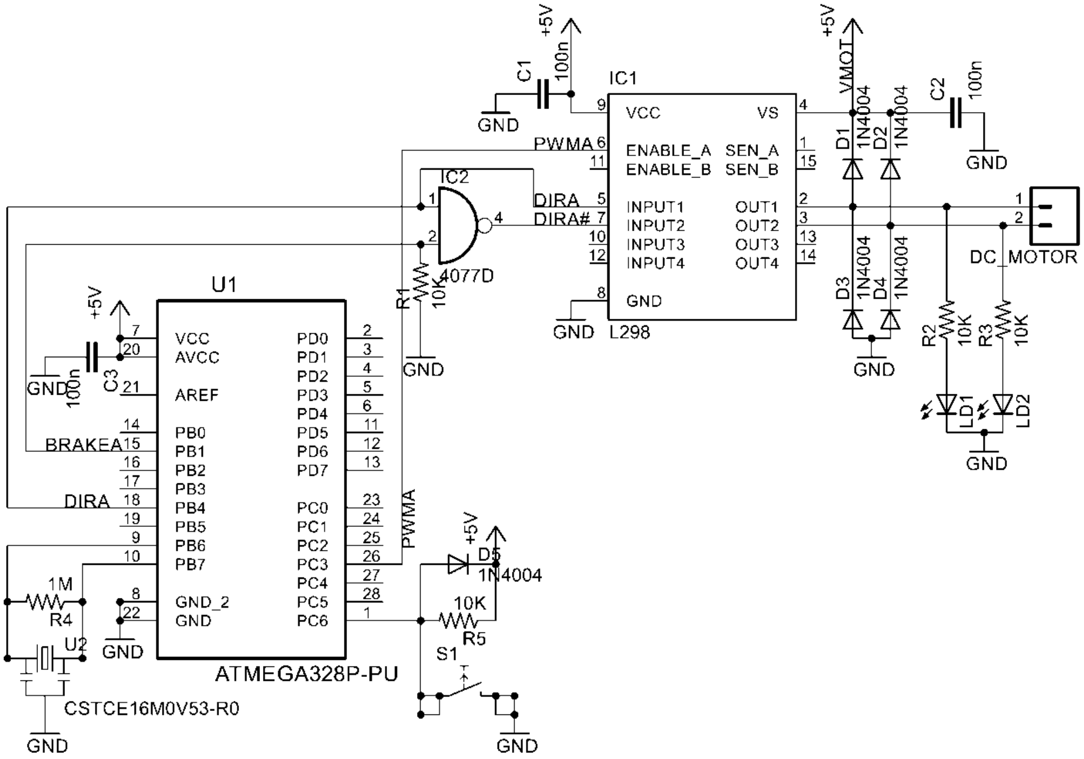

Figure 5S. Control circuit diagram of the spinning system.

<u>Operation of the spin system</u>

The spin system was designed to automate the mixing procedure in microfluidic disk. A DC motor (bridge A) starts to spin on clockwise direction at the speed of 1600 rpm for 4 seconds followed by ½ second brake engage state (stop rotation). Then the motor spins on the anti-clockwise directionat the speed of 1600 rpm for another 4 seconds followed by ½ second brake engage state (stop rotation). These four states of the DC motor were continuously repeated as long as the control circuit is powered. Figure 6S shows the program flowchart for the spinning

system. Although changes of the actual spin rate was not as rapid as the changes assigned in the microcontroller software, using a conventional DC motor has greatly reduced fabrication cost of the portable setup from hundreds of dollars to less than fifty dollars.

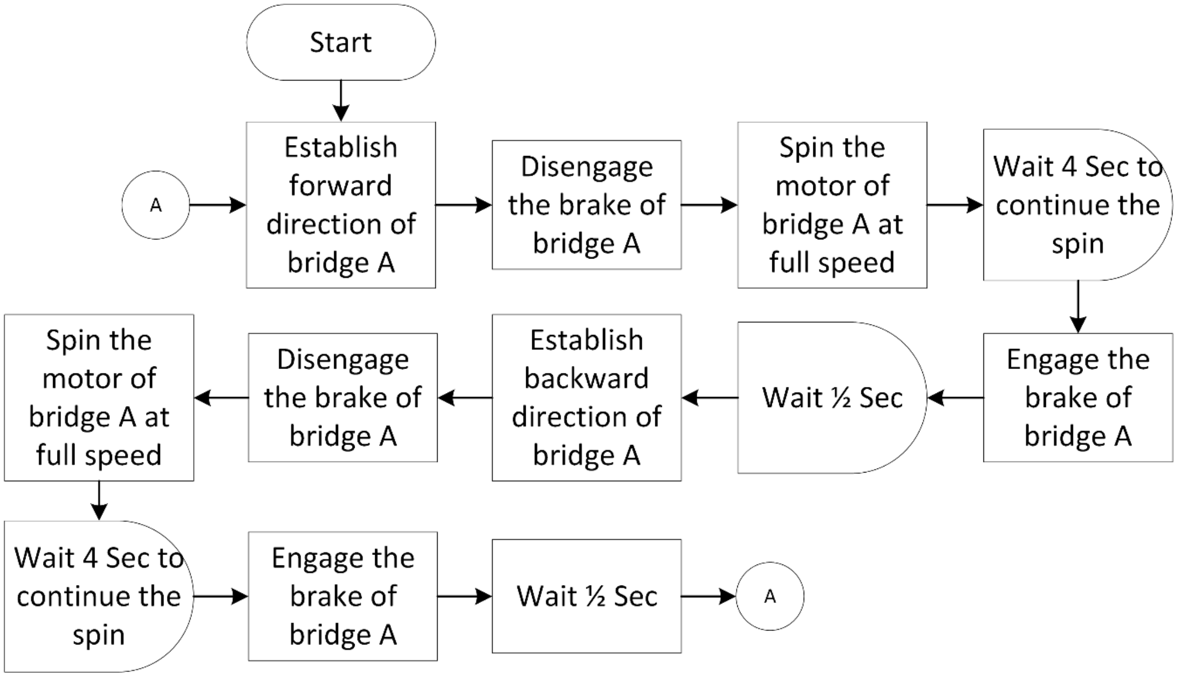

Figure 6S. Program flowchart of the spinning system.

Virus propagation in mosquito cells and titration

Dengue-infected cells with obvious cytopathic effects (CPE) were lysed by freeze and thaw cycle. The culture medium was centrifuged at RCF = 871 G for 10 min to remove cell debris, filtered (0.2 µm), portioned into aliquots, and stored at -80°C until used. The viral titter of the dengue suspension was established by serial dilutions on Vero cells using plaque assay. In brief, a 10-fold serial dilution of medium supernatant was added to new Vero cells grown in 24-well plate (1.5 x10^5 cells) and incubated for 1 h at 37°C. The cells were then overlaid with DMEM medium containing 1.1% methylcellulose. Viral plaques were stained with naphthol blue-black dye after 5 days of incubation. Virus titters were calculated according to the following formula:

titter (p.f.u/mL) = number of plaques / volume of diluted virus added to the well × dilution factor of the virus used to infect the well, in which plaques were enumerated. The titter of dengue virus that was used in the following experiments of colorimetric ELISA was 3.5×10^{7} p.f.u/mL and the serial dilutions were prepared in PBS.

Sandwich ELISA procedure

Step 1. Each well/microchamber of the analytical kit was charged with 100 µL of capture antibody (Ab), rabbit anti-dengue virus 2 antibody (ab155042, Abcam. US), which was diluted (1:500) in coating buffer (0.85 g of NaCl (Sigma. US), 0.14 g of Na_2HPO_4 (Sigma. US), and 0.02 g of NaH_2PO_4 (Sigma. US) in 100 mL of PBS (Fisher Scientific, Germany), pH= 7.4). Incubation was carried out for 2 hours at 37 °C.

Step 2. Washing step was performed with 200 µL per well/microchamber of washing buffer (0.05% Tween 20 (Sigma. US) in PBS, pH = 7.4) at room temperature. ELISA well plates and microfluidic disks of both, empty and including microspheres were washed 3 times (each time 5 minutes) by using shaker with the shaking speed of 1000 rpm. The exact same washing procedure was performed between each two steps of the ELISA assay.

Step 3. In order to achieve high selectivity and to avoid non-specific binding, blocking procedure was conducted by adding 100 µL of blocking buffer (1 g of BSA (Sigma. US) in 100 mL of washing buffer, pH = 7.4) to each well/microchamber. The incubation was carried out at 37°C for 1 hour.

Step 4. Each well/microchamber was charged with 100 µL of the virus solution and incubation was carried out for 2 hours at 37 °C. Different concentrations of the virus have been pre-determined depending on the application of the assay and virus solutions were prepared by serial dilution method.

Step 5. Primary Ab solution was prepared (1:200) by diluting mouse IgG2a anti DV (ab155863, Abcam. US) in diluting buffer (0.4 g of BSA, 4 mL of PBS buffer and 120 µL of Trintonx-100 in 36 mL of distilled water, pH = 7.4). Each well/microchamber has received 100 µL of primary Ab solution and was placed in the incubator for 2 hours at 37°C.

Step 6. The last incubation was conducted by adding 100 µL of anti-mouse igG2a alkaline phosphatase (ab97242, Abcam US) as secondary Ab, which was also diluted in diluting buffer (1:500). This step has been carried out at room temperature for duration of 30 minutes.

Step 7. Eventually wells/microchambers were thoroughly washed (as it was described in *step 2*) and charged with 100 µL of mixed substrate (alkaline phosphatase blue microwell substrate components A and B). The reaction was stopped after 15 minutes by adding 100 µL of alkaline phosphatase stop solution (A585, Sigma US) and signal intensity was recorded by using Bio-Rad (model 680) at the wavelength of 570 nm as the manufacture's guidelines suggested.

It should be noted that the time reduction, in the case of microfluidic disk, was applied in *steps 1, 4*, and *5* while *steps 2, 3, 6*, and *7* remained unchanged.

Negative controls

Negative controls for each individual system were calculated as a result of the assays, which were conducted with totally non-infected samples. Different dosages of the spheres applied in the assay as well as different incubation durations had minor impact on the resultant optical densities from the negative controls. Therefore, based on the plotted data in this section, representative negative controls have been depicted in the text to make the discussion clear.

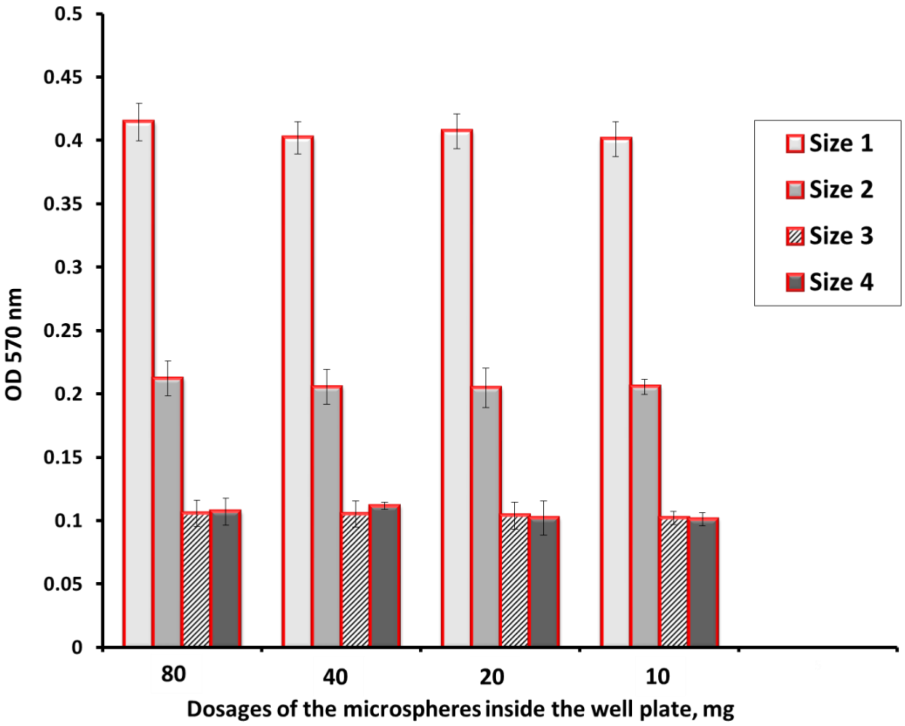

Figure 7S. Negative controls obtained from the assay that was conducted with different dosages of the spheres (from all size categories) in the absence of DV (spheres' dosage=20 mg).

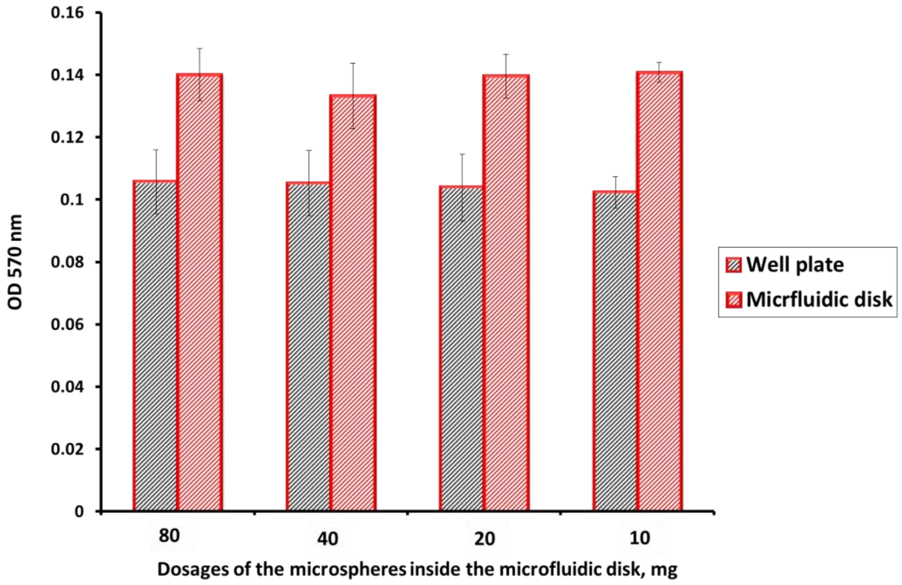

Figure 8S. Negative controls obtained from the assay that was conducted inside the well plate and microfluidic disk with selected size category of the spheres (size 3) in the absence of DV and variety of dosages of the spheres

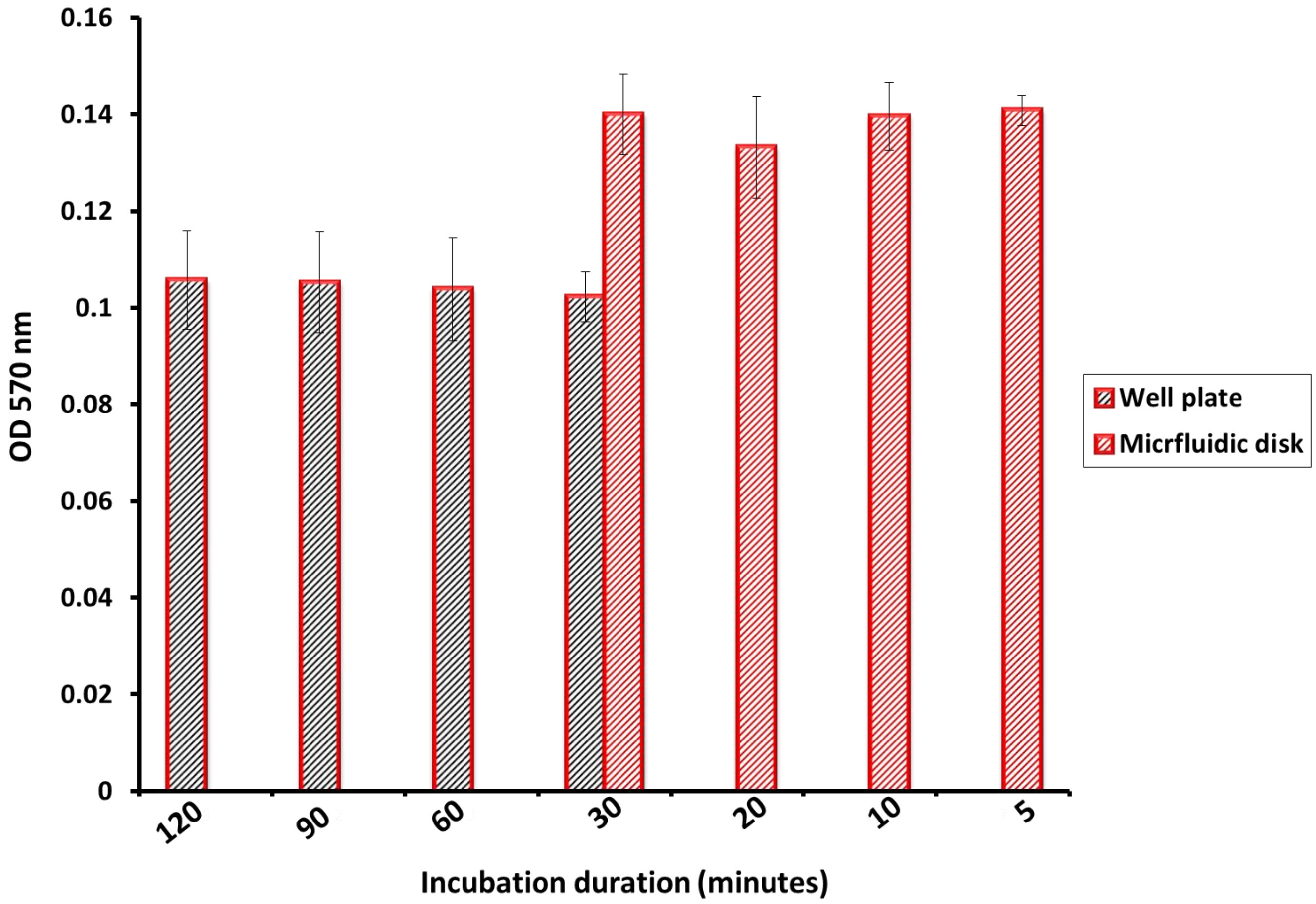

Figure 9S. Negative controls obtained from the assay that was conducted inside the well plate and microfluidic disk with selected size category of the spheres (size 3, 20 mg) in the absence of DV and in different incubation times.

Evaluation of analytical method

Sandwich ELISA was performed by using all of the size categories of the microspheres in different concentrations of DV (3.5×10^{-2} to 3.5×10^{-6} p.f.u/ml) in order to calibrate the assay and subsequently calculate the LoD values for the performed analytical method. From the plotted calibration curves (Fig. 10S) it was observed that higher level of reliability (average R^2=0.9478) was achieved from the generated detection signal by microspheres in comparison to conventional ELISA (R^2=0.9144). Among different sizes of the microspheres, size 3 provided the highest

precision of the detection (R^2=0.997) while the lowest level of reliability was received from the assay conducted with the smallest size of the microspheres (size 1, R^2=0.8651).

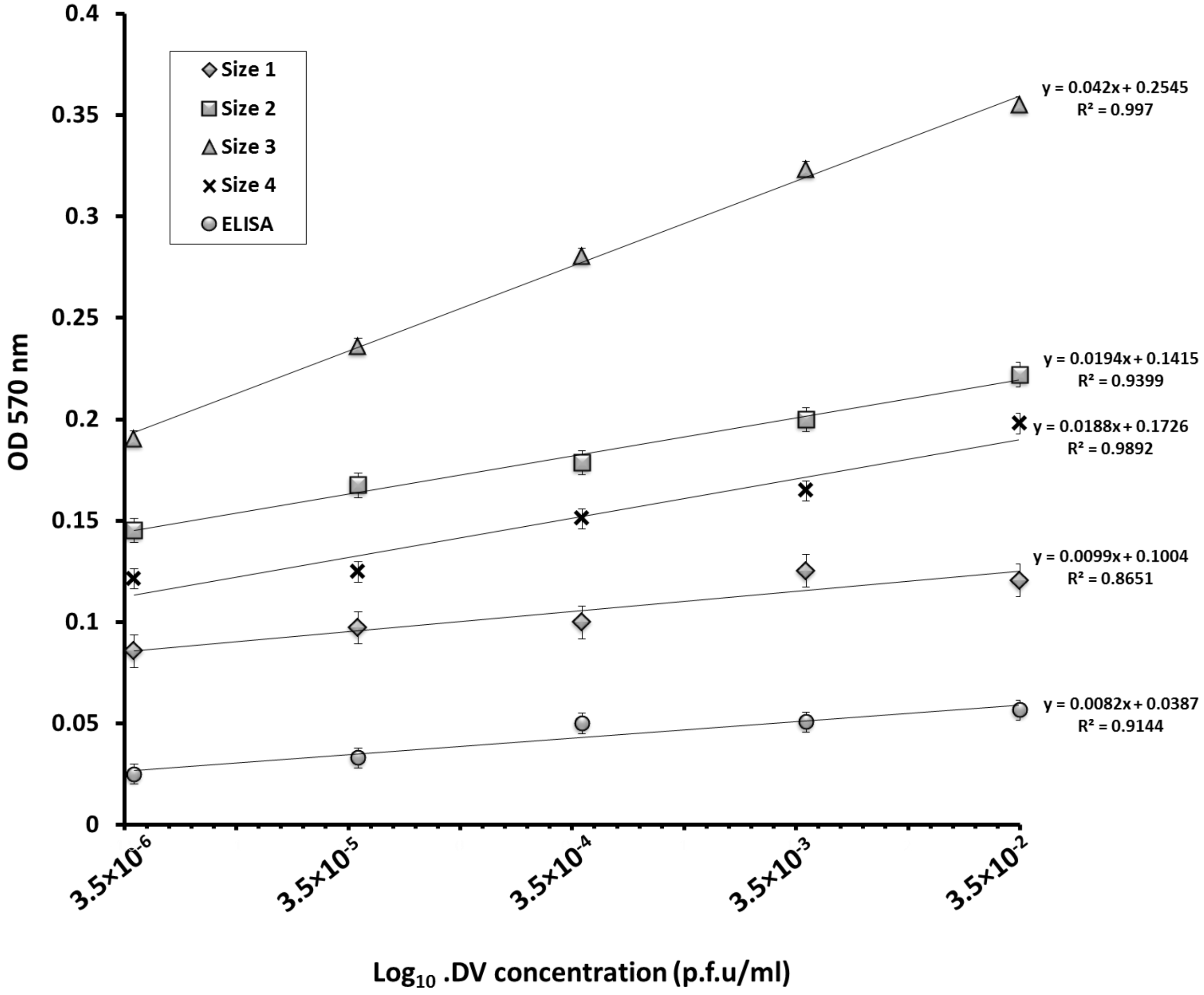

Figure 10S. Calibration curves obtained from sandwich ELISA assay performed with different concentrations of DV (3.5×10^{-2} to 3.5×10^{-6} p.f.u/ml) on microspheres of different sizes inside the well plate.

Table 1S provides calculated sensitivity and specificity of the proposed methodology by using microspheres of different sizes in sandwich ELISA. As it can be seen from Table 1S, satisfactory sensitivity results have been obtained for almost all of the micro-sized categories of the spheres.

Table 1S. Calculated sensitivity, specificity, accuracy and limit of detection (LoD) for microspheres in DV detection via sandwich ELISA.

DV status	Size 1		Size 2		Size 3		Size 4	
	+	-	+	-	+	-	+	-
Positive	56	6	61	3	63	1	62	2
Negative	8	10	3	13	1	15	2	14
Total	64	16	64	16	64	16	64	16
**Sensitivity (%)**	87.5		95.31		98.43		98.87	
**Specificity (%)**	62.5		81.25		93.75		87.5	
**Accuracy (%)**	82.5		92.5		97.5		95	
**LoD (p.f.u/mL)**	263		8.4		5.1		6.3	

However, specificity analysis indicated poor performance of size 1 microspheres as a result of frequent errors that occurred during the assay. Microspheres of size 3 have shown the supreme performance not only as the sensitive but also as the most specific platforms among all. In general, presented results in Table 1S suggest that developed microsphere (except size 1) are trustworthy bioreceptor in diagnostic systems. Moreover, accuracy of the assay for all of the developed platforms was calculated and results are shown in Table 1S as well. Significantly higher level of accuracy was achieved from different size categories of the microspheres in comparison to the conventional ELISA (evaluation of the conventional ELISA is presented in the text, Table1). Repeatedly, microspheres of size 3 have shown the highest level of accuracy (97.5%) among all the examined microspheres. Furthermore, calculated LoD values have proven the significant ability of the assay in biomolecular recognition. Detected concentrations of DV by

microspheres have remarkably reached to the lowest possible levels of approximately 4-8 p.f.u/mL, which is equivalent to the onset of the DF.

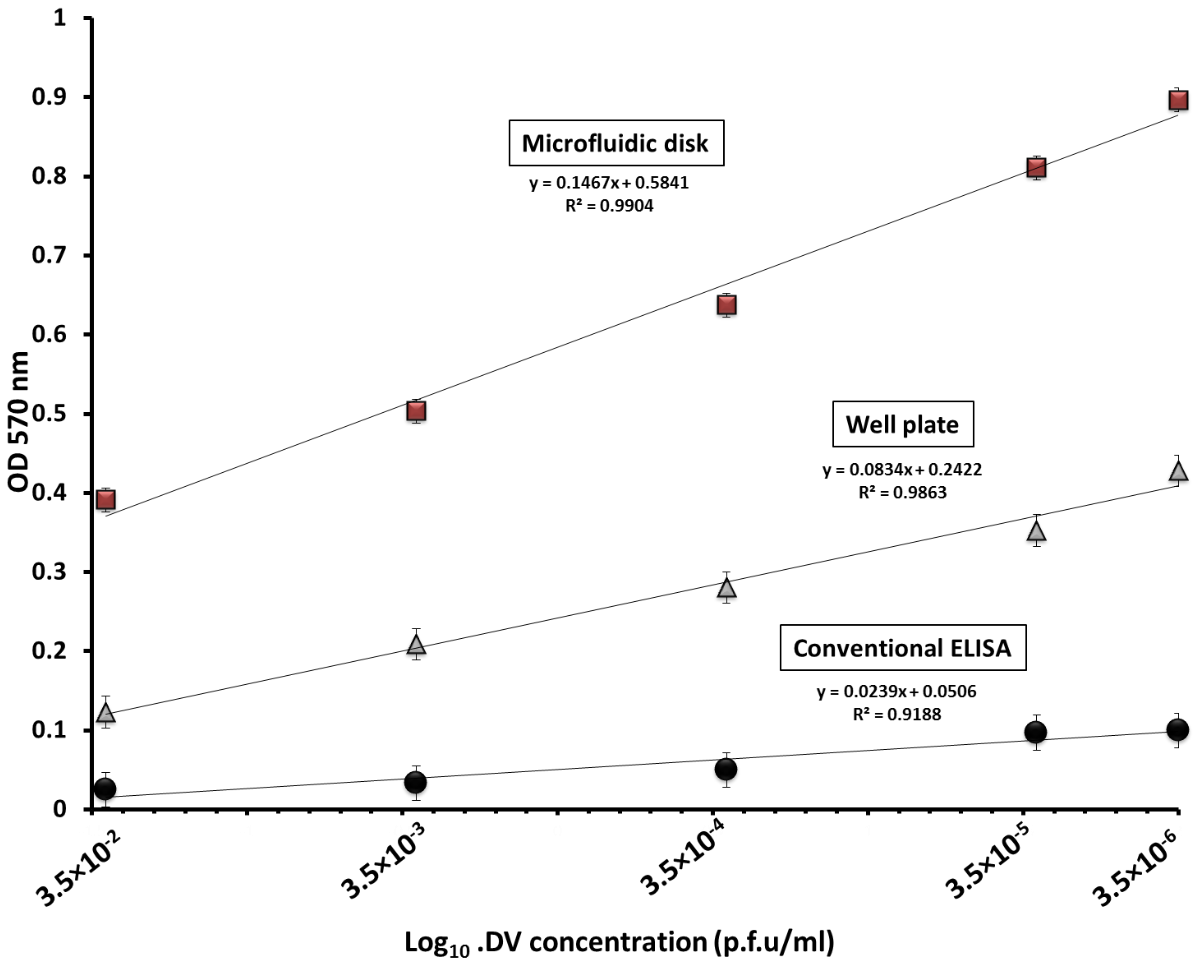

Figure 11S. Calibration curves obtained for different developed methodologies with integrated microspheres (size 3) in comparison to the conventional assay, ELISA. Noteworthy, calibration curves have been plotted by means of logarithm.

Same range of DV concentrations were utilized for plotting calibration curves and subsequent evaluation of developed systems (well plate and microfluidic disk in comparison to conventional ELISA). Resultant OD values were recorded at the specific wavelength (570 nm) and obtained data have been plotted against DV concentration. Calibration curves obtained from (i)

conventional ELISA; (ii) microspheres inside the well plates; and (iii) microspheres integrated into the microfluidic disk revealed increasing trend of reliability for mentioned examined systems, respectively. As it can be observed from Fig.11S, the square correlation coefficients (R^2) are significantly higher in the case of well plate (R^2= 0.9863) and microfluidic disk (R^2=0.9904) including microspheres (size 3) in comparison to what was obtained from conventional ELISA (R^2=0.9188). Presented calibration data are essential in determination of LoD values.
